# Possible high risk of transmission of the Nipah virus in South and South East Asia: a review

**DOI:** 10.1186/s41182-023-00535-7

**Published:** 2023-08-10

**Authors:** Jagadish Joshi, Yogendra Shah, Kishor Pandey, Ram Prashad Ojha, Chet Raj Joshi, Lok Raj Bhatt, Shyam Prakash Dumre, Pushpa Raj Acharya, Hem Raj Joshi, Shikha Rimal, Ramesh Shahi, Deepak Pokharel, Kamal Singh Khadka, Bimal Dahal, Saroj Nepal, Ram Singh Dhami, Krishna Prasad Pant, Rajdip Basnet, Basu Dev Pandey

**Affiliations:** 1 Health Directorate, Doti, Rajpur, Sudurpaschim Province Nepal; 2 Province Public Health Laboratory, Rajpur, Kailali Nepal; 3 Everest International Clinic and Research Center, Kathmandu, Nepal; 4 Central Department of Zoology, Tribhuvan University, Kirtipur, Nepal; 5 Ministry of Social Development, Kailali, Dhangadhi, Sudaurpaschim Province Nepal; 6 Central Department of Microbiology, Tribhuvan University, Kirtipur, Nepal; 7 Central Campus of Science and Technology, Faculty of Science, Mid-West, University, Surkhet, Nepal; 8 Himalayan College of Agricultural Sciences and Technology (HICAST), Kirtipur, Nepal; 9 Seti Provincial Hospital, Kailali, Dhangadhi, Nepal; 10 Department of Microbiology, Janapriya Multiple Campus, Kaski, Pokhara, Nepal; 11 Department of Microbiology, Trichandra Multiple Campus, Kathmandu, Nepal; 12 Department of Microbiology, Far Western-University, Bhimdutta, Nepal; 13 Central Department of Biotechnology, Tribhuvan University, Kirtipur, Nepal; 14 DEJIMA Infectious Disease Research Alliance, Nagasaki University, Nagasaki, Japan

**Keywords:** Nipah virus, Southeast Asia, ELISA, PCR, One Health

## Abstract

Nipah virus (NiV) is a zoonotic, single-stranded RNA virus from the family *Paramyxoviridae,* genus *Henipavirus*. NiV is a biosafety-level-4 pathogen that is mostly spread by *Pteropus* species, which serve as its natural reservoir host. NiV is one of the major public health challenges in South and South East Asia. However, few molecular studies have been conducted to characterise NiV in a specific region. The main objective of this review is to understand the epidemiology, pathogenesis, molecular surveillance, transmission dynamics, genetic diversity, reservoir host, clinical characteristics, and phylogenetics of NiV. South and South East Asian nations have experienced NiV outbreaks. Phylogenetic analysis confirmed that two primary clades of NiV are in circulation. In humans, NiV causes severe respiratory illness and/or deadly encephalitis. NiV is mainly diagnosed by ELISA along with PCR. Therefore, we recommend that the governments of the region support the One Health approach to reducing the risk of zoonotic disease transmission in their respective countries.

## Introduction

### Nipah virus

**Fig. 1:**
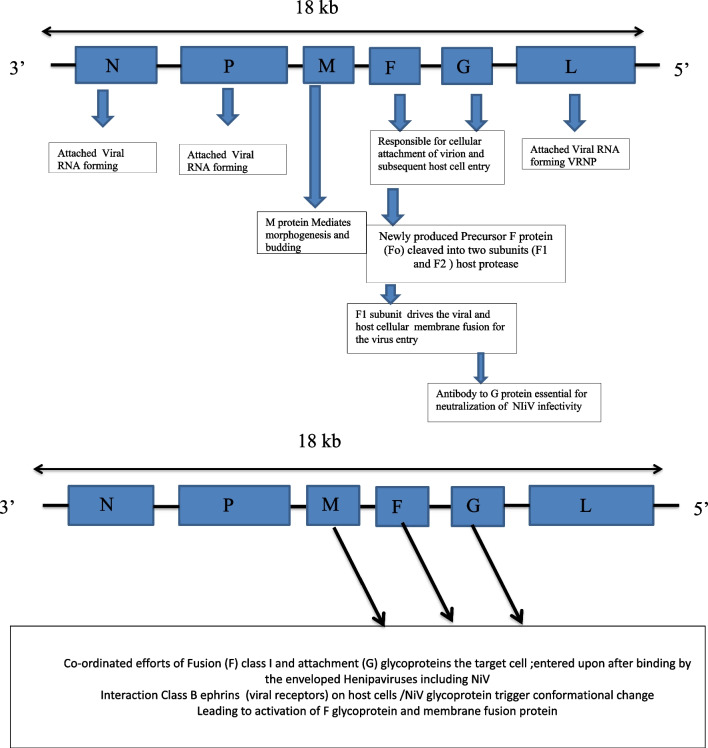
Structure of NiV and Pathogenesis of NiV infection Source: References [6–11]

Nipah virus (NiV) disease is an emerging zoonotic disease. NiV is an RNA virus from the family *Paramyxoviridae,* genus *Henipavirus*, subfamily *Paramyxovirinae* and order Mononegavirales, causing severe respiratory disease and encephalitis in South Asia, with high mortality [1, 2]. NiV closely resembles the Hendra virus, Cedar virus and Mojiang virus [3]. NiV is pleomorphic, spherical, thread-like and enveloped, with a size of 40–1900 nm, negative-sense, non-segmented, with a single-stranded RNA possessing helical symmetry with an average length of approximately 18 kb [44]. The RNA genome of NiV is composed of six major structural proteins: nucleocapsid (N), phosphoprotein (P), matrix (M), fusion glycoprotein (F), attachment glycoprotein (G) and long polymerase (L). Furthermore, N, P and L are responsible for the attachment of viral RNA to form the virus ribonucleoprotein (vRNP), whereas F and G are responsible for the cellular attachment of the virion and the subsequent entry into the host cell [6–13] (Fig. 1).

Phylogenetic analysis genetically characterised NiV into two major distinct genotypes: NiV-Malaysia (NiV-MY), recognised in Malaysia and Cambodia, and NiV-Bangladesh (NiV-BD), recognised in Bangladesh and India [14, 15]. Furthermore, NiV-MY genotypes have shown more virulence than NiV-BD, which directly correlates with the clinical manifestation, pathogenicity, route of transmission and severity of the disease [16].

The reservoir hosts of NiV are fruit bats belonging to the genus *Pteropus*, also called flying foxes [17]. Sequencing data suggests that NiV is most closely related to the Hendra virus and evolutionary originated from the route of bat species. A sero-epidemiological study from Malaysia showed that four fruit bat species were implicated: *Pteropus hypomelanus*, *Pteropus vampyrus*, *Cynopterus brachyotis* and *Eonycteris spelaea,* as well as the insectivorous bat *Scotophilus kuhlii* [18]. Likewise, the fruit bat species *Pteropus giganteus*, *Pteropus lylei* and *Hipposideros larvatus* were also verified as reservoir hosts of NiV in Bangladesh, Cambodia and Thailand [19]. Serological tests based on antibodies against the NiV detected its presence in the serum of fruit bats collected in India, Indonesia and Timor-Leste [20]. Likewise, epidemiological studies on bat species from Nepal (*Pteropus giganteus, Rousettus leschenaultii, Eonycteris spelaea, Rhinolophus sinicus, R. affinis, R. ferrumequinum, Nyctalus noctula and Scotophilus sp.*) reported that potential reservoir hosts for several viruses are often found in human proximity and some of them even enter human dwellings at night [21]. Some fruit bats such as *P. giganteus* and *R. leschenaultii* are even consumed as bushmeat by some communities in Nepal [21]. Nepal also faces a high risk of rabies virus transmission from bat bites, implying that a zoonotic viral outbreak is likely in the future [21, 22]. However, the situation of NiV infections in other countries in South and South East Asia is unknown.

NiV encephalitis is an emerging and re-emerging infectious disease considered a major public health problem by the World Health Organization (WHO). NiV is present mainly in South East Asia and can cause severe respiratory illness and deadly encephalitis in humans. NiV can be transmitted to humans from animals (bats or pigs), contaminated foods or infected fluids and can also be transmitted directly from human to human. The main objective of this review is to understand the epidemiology, pathogenesis, molecular surveillance, transmission dynamics, genetic diversity, reservoir host, clinical characteristics, and phylogenetics of NiV.

## Methods

A literature search was performed using PubMed, Web of Science and Google Scholar. The following keywords were used: ‘Nipah virus infection’ or ‘epidemiology Nipah virus’, or ‘clinical features Nipah virus’ or ‘diagnosis Nipah virus’, or ‘surveillance Nipah virus’, or ‘prevention and control’, ‘South and South East Asia’. Research papers, review papers and case reports were included in the search.

## Epidemiology and Nipah virus outbreaks (1998–2020)

NiV was named after the village of Sungai Nipah (Nipah River Village) in Negeri Sembilan, Malaysia, where NiV was first identified in 1999. At that time, the virus crossed the species barrier from bats and infected humans, resulting in encephalitis with up to 40% mortality and residual neurological problems among survivors. The outbreak was first recognised in pigs that consumed fruit partially eaten by fruit bats and transmitted the infection to humans. In March 1999, an outbreak of acute NiV infection was confirmed in 11 male abattoir workers with an average age of 44 years in Singapore. One of these workers died because of speculated transmission of the virus during the import of pork from Malaysia. Between 1998 and 1999, both countries (Singapore and Malaysia) reported 246 cases of febrile encephalitis by NiV in addition to pigs in farms showing neurological and respiratory symptoms. In 2004, Bangladesh confirmed its first NiV outbreak by the presence of anti-NiV antibodies in serum samples. Since 2004, researchers have detected the viral nucleic acid of NiV by genome analysis. From 2004 to 2010, nine outbreaks were documented in Bangladesh. Likewise, in 2011 another NiV outbreak was reported to cause 15 deaths in a remote town named Hatibandha in the Lalmonirhat district of northern Bangladesh [23]. The research findings showed that the main source of NiV transmission in Bangladesh was the consumption of infected raw date palm fruits [24] (Table 1).

**Table 1:** Nipah Virus Outbreaks in South Asia Countries (1999–2021). Sources: updated from WHO–SEARO References [35–40]

Country (place)	Year	No. of cases	No. of deaths (Case fatality rate, %)	Primary route of transmission	Laboratory diagnosis
Malaysia	1998–1999	265	105(39.6%)	Close contact with pigs (i.e., pig farmers)	
Singapore	1999	11	1(9.1%)	Contact with pigs	
India (Siliguri)	Jan–Feb 2001	66	45(68.2%)	Human to human close direct contact Contact with bats from the Pteropus spp.	
Bangladesh	Apr–May 2001	13	9(69.2%)	Consumption of fruits or fruit products (raw date palm juice) - Contaminated with urine or saliva from infected fruit bats	
Bangladesh	Jan 2003/April 2004	12/31	8/23(67%/74%)	Same as above	
Bangladesh	Jan–Mar 2005	12	11(9%2)		
Bangladesh	Jan–Feb 2007/March–April 2007	8/3	5/1(63%/33%)		
India (Nadia)	Apr 2007	5	5(100%)		
Bangladesh	Feb–April 2008	4/7	4/5(100%/71%)		
Bangladesh	Jan 2009	4	1(75%)		
Bangladesh	Feb–March 2010/Jan–Feb 2011	16/44	14/40(88%/91%)		
Bangladesh	Feb 2012/Jan–Apr 2013	12/24	10/21(83%/88%)		
Philippines	2014	17	9(53%)		
Bangladesh	2014/2015	18/9	9/6(50%/67%)		
India (Kerala State)	May 2018	18	17(94.4%)	Suspected of consumption of fruits or fruit products	
India (Kerala State)	September 2021	1	1(100%)	Contact with horses/Consumption of horse meat	On 4 September, 2021 the presence of Nipah virus in the plasma, cerebrospinal fluid and serum samples was confirmed by real-time polymerase chain reaction (RT-PCR) and IgM antibodies was confirmed in the plasma sample by ELISA serology test at NIV Pune

The first outbreak of NiV in India was detected in Siliguri, West Bengal, in 2001, characterised by febrile illness in association with altered sensorium [25]. Some Siliguri outbreak isolates showed close similarity to those of the outbreak in Bangladesh. A subsequent outbreak was confirmed in Nadia, West Bengal, in 2007. Then, in 2018, a NiV infection outbreak was reported in Kozhikode, northern Kerala, India, with fruit bats as the source of the outbreak [26, 27]. During the 2018 outbreak, most deaths occurred in infected patients and health personnel who were involved in their treatment. A total of 60 infected persons died in the Malappuram and Kozhikode districts, identified by laboratory diagnosis employing real-time polymerase chain reaction (RT-PCR). DNA sequencing showed that the genome of the confirmed isolates of NiV infection was very similar to the BD strain of MY [28]. Recently, a fifth NiV outbreak on 4 September 2021 was reported by the Kerala State Health Department in Kozhikode, Kerala, India. On 29 August 2021, a 12-year-old boy showed a low-grade fever and was transferred to several hospitals but his condition deteriorated. On 4 September 2021, the presence of NiV in the boy’s plasma, cerebrospinal fluid and serum samples was confirmed by RT-PCR, and IgM antibodies were detected in the plasma sample by ELISA. The next day, the patient died and a safe cremation was performed on the same day in Kozhikode. Since then, NiV infection has claimed at least 20 lives and is rapidly spreading in the southern state of Kerala [28] (Table 1).

NiV infection in humans has a range of clinical presentations from asymptomatic infection to acute respiratory infection, fatal encephalitis and severe neurological disease. The death rate is in the range of 40–75% according to reported outbreaks between 1998 and 2018 [29–31]. According to the WHO, NiV is one of the highly infectious pathogens that should be handled in facilities classified at biosafety level 4 (BSL4), which is challenging because of the limited access to sophisticated laboratories in most countries.

**Fig. 2:**
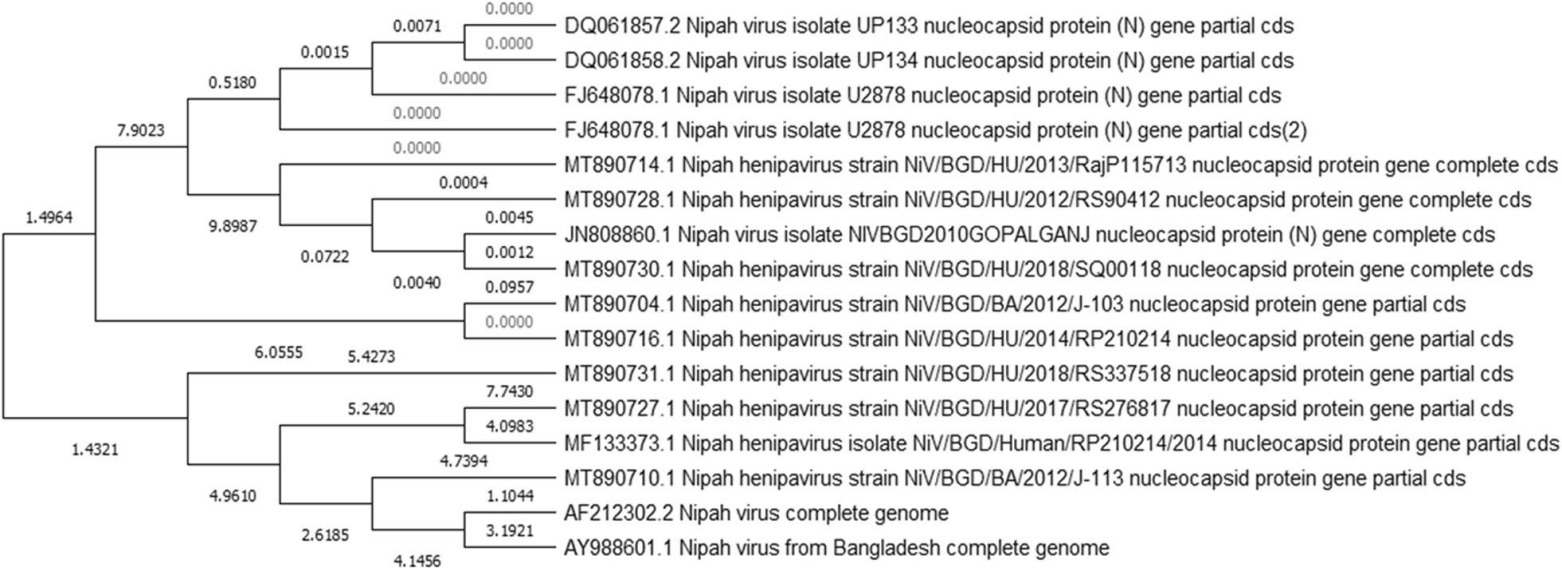
Phylogenetic analysis of NIV infection in South Asia Countries (1999–2021). Source: References [35–40]

A study on the genetic diversity of NiV in Bangladesh was conducted in 2012. RT-PCR was used to screen a total of 456 bat roost urine samples; among them, 38 (8%) were positive for NiV RNA, with cycle threshold (CT) values ranging from 32.7 to 38.5 including NiV-N gene fragments (N ORF 188–1599) sequenced from 100 roost urine specimens [32]. Moreover, in a follow-up study, 39 samples were collected from humans during 2012–2018 in areas, where NiV RNA was previously detected. RNA was detectable in 31 samples (80%) by RT-PCR, and nucleotide sequences were successfully identified from 21 samples by Sanger sequencing [32]. Phylogenetic tree analyses revealed that the N gene sequence obtained from bat roost urine in Joypurhat in 2012 was almost identical (98.6–95.4%) to the NiV-BD genotype present in Bangladesh, India and Thailand. The phylogenetic tree revealed an evolutionary rate of 4.64 × 10^4^ for the NiV-BD genotype, which diverged from 1995 into two sub-lineages. Furthermore, phylogenetic tree analyses from bat roost urine and human samples categorised these samples into the same genotype (NiV-BD). NiV sequences from Malaysia, Cambodia and Thailand were categorised into NiV-MY genotypes, whereas those from Bangladesh, India and Thailand were categorised into other NiV-BD genotypes (Fig. 2). The above studies [32] provide molecular information for designing laboratory methods for the early detection of NiV in environmental samples to evaluate its potential risk of transmission in the human population. More detailed studies on the genetic variability between the NiV strains detected in bats and humans can also contribute to identifying the transmissibility and infection potential of the virus and can thus help to predict outbreaks and identify vaccine–candidate strains.

Nepal is a South Asian country located along the central foothills of the Himalayas, sharing porous borders with India in the south, east and west. Open borders can cause an unprecedented outbreak of NiV in Nepal through human transmission. Furthermore, some fruit bat colonies in the foothills of Nepal are speculated to adopt seasonal migration patterns from higher to lower elevations (cross-border migration as well), which represents a high risk of zoonotic virus spread in Nepal. Another important factor that contributes to the rapid transmission of infectious diseases in South Asian and South East Asian countries is the increase in the human population [33]. Poultry and pig farming and cattle rearing in the proximity of fruit bat colonies may increase the risk of transmission of zoonotic diseases. Bats generally prefer warmer temperatures, although a few bat species can live in cold environments through torpor and hibernation. Hence, in the mountains of Nepal, some bats roost in human-made structures to avoid the cold in winter. Because bats co-exist with human communities, the risk of a bat-borne viral outbreak in Nepal is latent, although there is no solid evidence of such a health hazard for the country. Our emphasis is on preparedness to tackle such probable risks by increasing our understanding of the disease based on research and solid evidence. Therefore, the authorities in South Asian countries need to remain vigilant to tackle NiV outbreaks [34].

Many studies have confirmed that fruit bat species (*Pteropus*) are the natural hosts of NiV, which is a virus of concern for future epidemics and also seems to be spilling over from its animal reservoir to humans. Sequencing analysis confirmed that fruit bats are responsible for the transmission of NiV to domestic animals and humans through contaminated food or indirectly from person to person. Thus, NiV infection in domestic animals such as pigs should be monitored routinely and the farm should be cleaned daily using appropriate disinfection agents or detergents to prevent an outbreak. At border checkpoints in South Asian countries, domestic animals should be quarantined and tested for zoonotic diseases. In addition, banning the movement of animals from infected farms to other areas can reduce the spread of disease. Moreover, the One Health approach should be strictly employed to detect new cases by performing surveillance and quarantining domestic animals for providing an early warning to veterinary and human public health authorities [34–39].

## Laboratory diagnosis and treatment

There are currently no specific vaccines or effective antiviral drugs available for the treatment of NiV infection. However, intensive and supportive care and prevention will be the mainstays of management, as treatments are only recommended by the WHO in the case of severe respiratory and neurologic complications. NiV infection can be diagnosed at the early stages through RT-PCR tests of the throat, nasal passage, urine, cerebrospinal fluid and blood. Furthermore, diagnosis may also include nucleic acid amplification, sequencing, immunofluorescence assay, histopathology, virus isolation, neutralisation and high throughput techniques for whole-genome sequencing of NiV infecting humans and animals. In addition, viruses can be diagnosed during the patient’s recovery stage, and antibodies can be detected using IgG/IgM ELISA. According to the WHO and standard diagnostics for NiV, PCR is strongly recommended as the most sensitive method for diagnosis of active NiV infection but NiV-specific IgM ELISA is an alternative serological approach, where PCR is not available. However, although the detection of NiV with ELISA is reliable, it has less sensitivity and specificity than molecular detection [34–42].

## Prevention of NiV

Scientists are currently working on the development of antiviral drugs and vaccines for NiV. The National Institute of Allergy and Infectious Diseases conducted a clinical trial of an mRNA NiV agent. For the prevention and control of NiV in South and South East Asia, each country in the region should develop an action plan including policies and strategies for controlling the virus. This should be followed by the implementation of the endorsed action plan strategies and raising public health awareness regarding NiV outbreaks and the importance of preventive measures to mitigate risk factors. In the twenty-first century, social media platforms are one of the most important modes used for informing the public in addition to local television, FM radio stations and printed media (e.g., posters and pamphlets). For instance, farm workers may be instructed to avoid direct contact with farm animals, especially during slaughter and disposal, and wear appropriate personal protective equipment and clothing. Villagers may be taught to avoid consuming date palm fruits and drinking date palm juice that may be contaminated if bats live on those palm trees. Furthermore, surveillance of NiV and other infectious diseases that could be at high risk of transmission from animals to humans should be carried out. High-risk areas in South and South East Asia along the NiV girdle should be identified and mapped, and then continual surveillance should be conducted to facilitate the early detection of virus outbreaks, identify circulating strains among fruit bats, and monitor environmental factors and the dynamics of wide-ranging transmission.

To respond to an outbreak, specialised research teams should be established to identify suspected cases of infection in humans and further analyse the possibility of the emergence of new species as potential reservoir hosts of the virus that may be responsible for its transmission to humans from bats. In other words, establishing mechanisms for cross-border screening, laboratory testing, and diagnosis facilities at points of entry and exit would ensure that suspected cases are referred to facilities at the provincial level for confirmation of NiV. Furthermore, more reliable tests for the surveillance of communities and livestock will be vital to achieving a better understanding of the ecology of the fruit bat host and transmission risks to intermediate hosts, enabling the implementation of the One Health approach for outbreak prevention and the management of zoonotic diseases like that caused by NiV. In summary, the control and prevention of NiV would be possible only if strictly endorsed guidelines and control strategies are implemented by employing the One Health approach in all South and South East Asian countries [4, 5, 40, 43, 44].

## Role of medicinal plants for NiV treatment

In the absence of specific antivirals and vaccines, medicinal plants can play an important role by providing therapeutic agents for the treatment of various viral diseases. Previous studies have revealed that medicinal plants have promising antiviral properties and are used to protect against various public health threats that affect humans and animals. South East Asian countries have medicinal plant candidates that could be screened for the treatment of viral diseases, such as NiV. The following steps would be taken for this purpose: evaluation of current treatment approaches for viral infection, screening of antiviral drug candidates from natural products and elucidation of mechanisms, inhibition of viral protease, viral replication, viral spike protein, and receptor, and finally, screening of medicinal plant target compounds to develop an antiviral drug against NiV. The best method will probably be the screening of compounds that are responsible for altering or disturbing any step of the virus’ replication. Studies on medicinal plants for the treatment of viral diseases have reported 93 antiviral drug candidates, which could be a potential area of research in antiviral drug discovery. Furthermore, these drug candidates might also show inhibitory effects against influenza, parainfluenza, and other respiratory viruses [45]. Therefore, medicinal plant extracts, phytochemicals, and herbs could have potential as antiviral agents for the effective control of NiV, and medicinal plant formulations could prevent or cure NiV and other highly infectious pandemic-prone viral diseases in Asia [46]. In-depth studies will be necessary, using both in vitro and in vivo models, to test the medicinal plants and their phytochemicals to measure their antiviral activities against NiV infection [45, 46].

## Recommendations

South Asian and South East Asian countries should increase awareness of the NiV risk factors and educate their people on measures to reduce NiV exposure and risk of infection. By applying the strategies listed in [39], public health educational messages should focus on reducing the following risk factors: bat-to-human transmission, animal-to-human transmission and human-to-human transmission. Healthcare workers should be aware of this risk while caring for suspected or confirmed patients or handling specimens, and should strictly follow the universal standard infection prevention guidelines (i.e., wearing personal protective equipment, an N95 mask, goggles and a face shield) at all times. Furthermore, samples from people and animals with suspected NiV infection should be collected and handled by trained health and laboratory personnel in a well-equipped molecular laboratory. Standard guidelines should be followed at all times when handling suspected or confirmed animal or human samples. Effective vaccination strategies ought to be developed in the nearby future to tackle the risk of infectious agents, such as NiV. Therefore, we encourage the governments of South Asian and South East Asian countries to endorse the One Health approach to reduce the risk of zoonotic disease transmission [35–42]. For instance, India has adopted guidelines for the clinical management of NiV disease, using the One Health approach. Moreover, the Indian Council of Medical Research and Integrated Disease Surveillance Programme has partnered with veterinary divisions, such as the National Centre for Disease Control in India. This model could be emulated by other South Asian nations [47, 48].

## Data Availability

The information on the Nipah virus in South and South East Asiafrom references [35–40]

## Abbreviations

NiV: Nipah virus
ELISA: Enzyme-linked Immunosorbent Assay
PCR: Polymerase Chain Reaction
RT-PCR: Real-Time Polymerase Chain Reaction
WHO: World Health Organization
